# Characterisation and antimicrobial activity of biosurfactant extracts produced by *Bacillus amyloliquefaciens* and *Pseudomonas aeruginosa* isolated from a wastewater treatment plant

**DOI:** 10.1186/s13568-017-0363-8

**Published:** 2017-05-31

**Authors:** Thando Ndlovu, Marina Rautenbach, Johann Arnold Vosloo, Sehaam Khan, Wesaal Khan

**Affiliations:** 1 Department of Microbiology, Faculty of Science, Stellenbosch University, Private Bag X1, Stellenbosch, 7602 South Africa; 2 BIOPEP Peptide Group, Department of Biochemistry, Faculty of Science, Stellenbosch University, Private Bag X1, Stellenbosch, 7602 South Africa; 3 Faculty of Health and Applied Sciences, Namibia University of Science and Technology, 13 Storch Street, Private Bag 13388, Windhoek, Namibia

**Keywords:** *Bacillus amyloliquefaciens* ST34, *Pseudomonas aeruginosa* ST5, Surfactin, Rhamnolipid, UPLC–MS, ESI–MS

## Abstract

Biosurfactants are unique secondary metabolites, synthesised non-ribosomally by certain bacteria, fungi and yeast, with their most promising applications as antimicrobial agents and surfactants in the medical and food industries. Naturally produced glycolipids and lipopeptides are found as a mixture of congeners, which increases their antimicrobial potency. Sensitive analysis techniques, such as liquid chromatography coupled to mass spectrometry, enable the fingerprinting of different biosurfactant congeners within a naturally produced crude extract. *Bacillus amyloliquefaciens* ST34 and *Pseudomonas aeruginosa* ST5, isolated from wastewater, were screened for biosurfactant production. Biosurfactant compounds were solvent extracted and characterised using ultra-performance liquid chromatography (UPLC) coupled to electrospray ionisation mass spectrometry (ESI–MS). Results indicated that *B.* *amyloliquefaciens* ST34 produced C_13–16_ surfactin analogues and their identity were confirmed by high resolution ESI–MS and UPLC–MS. In the crude extract obtained from *P. aeruginosa* ST5, high resolution ESI–MS linked to UPLC–MS confirmed the presence of di- and monorhamnolipid congeners, specifically Rha–Rha–C_10_–C_10_ and Rha–C_10_–C_10_, Rha–Rha–C_8_–C_10_/Rha–Rha–C_10_–C_8_ and Rha–C_8_–C_10_/Rha–C_10_–C_8_, as well as Rha–Rha–C_12_–C_10_/Rha–Rha–C_10_–C_12_ and Rha–C_12_–C_10_/Rha–C_10_–C_12_. The crude surfactin and rhamnolipid extracts also retained pronounced antimicrobial activity against a broad spectrum of opportunistic and pathogenic microorganisms, including antibiotic resistant *Staphylococcus aureus* and *Escherichia coli* strains and the pathogenic yeast *Candida albicans*. In addition, the rapid solvent extraction combined with UPLC–MS of the crude samples is a simple and powerful technique to provide fast, sensitive and highly specific data on the characterisation of biosurfactant compounds.

**Electronic supplementary material:**

The online version of this article (doi:10.1186/s13568-017-0363-8) contains supplementary material, which is available to authorized users.

## Introduction

Biosurfactants are secondary metabolites that are non-ribosomally synthesised by actively growing and/or resting microbial cells (bacteria, fungi and yeast) (Van Delden and Iglewski 1998; Ron and Rosenberg 2001; Mulligan 2005). They have been classified into different groups based on their chemical composition and microbial origin and they are divided into five major classes which include glycolipids, lipopeptides, phospholipids, polymeric compounds and neutral lipids (Ron and Rosenberg 2001; Sen 2010). While they have been extensively applied in bioremediation, industrial emulsification and enhanced oil recovery (Banat et al. 2014), certain biosurfactant compounds have also been reported to display multipurpose biomedical and therapeutic properties, which include applications as antiadhesives, anticarcinogens and antimicrobials (Benincasa et al. 2004; Mulligan 2005; Rodrigues et al. 2006; Mulligan et al. 2014).

Glycolipids and lipopeptides constitute the most widely studied groups of biosurfactant compounds displaying broad spectrum antimicrobial activity and are currently applied in several fields (cosmetic, food and pharmaceutical industries) as antimicrobial, emulsifying and surfactant agents (Mandal et al. 2013). The glycolipid based biosurfactants include mannosylerythritol lipids, sophorolipids, trehalolipids and the most dominant group rhamnolipids, that are primarily produced by *Pseudomonas* species, particularly *P. aeruginosa* strains. Rhamnolipids consist of one or two rhamnose residues in their hydrophilic moiety linked to one, two or three hydroxyl fatty acid chains of varying lengths (eight to 22 carbons) (Déziel et al. 1999; Gunther et al. 2005).

The lipopeptides generally contain similar peptide chains (short linear or cyclic structures). The hydrophilic moiety is composed of amino acid residues varying only at specific residues and is linked to varying lengths (saturated and unsaturated) of fatty acids that act as the hydrophobic moiety (Makovitzki et al. 2006; Raaijmakers et al. 2010; Yao et al. 2012; Mandal et al. 2013). Lipopeptides are widely produced by *Bacillus* species and they consist of bacillomycins, fengycins, iturins, mycosubtilins as well as the widely studied lipopeptide, surfactin (Ongena and Jacques 2008; Raaijmakers et al. 2010; Sansinenea and Ortiz 2011; Chen et al. 2015; Inès and Dhouha 2015). Surfactin is a cyclic heptapeptide consisting of hydrophobic and negatively charged amino acids with a chiral sequence LLDLLDL linked to hydroxyl fatty acyl residue of between 12 and 16 carbon atoms (Seydlová and Svobodová 2008).

Several isoforms and analogues exist for the naturally produced glycolipids and lipopeptides, which is why they exhibit significant structural heterogeneity (Benincasa et al. 2004; Ongena and Jacques 2008). A variety of methods are utilised to classify and characterise the biosurfactant compounds produced by a range of microorganisms. Mass spectrometry (MS) coupled with various chromatographic methods are the most widely used techniques, where liquid chromatography (LC) coupled to electrospray ionisation mass spectrometry (ESI–MS) and matrix-assisted laser desorption ionisation time-of-flight mass spectrometry (MALDI–TOF–MS) have shown a high sensitivity and accuracy in various analyses. MALDI–TOF–MS analysis enables the rapid fingerprinting of low concentrations of metabolites directly from actively growing/resting microbial cells (Bright et al. 2002; Singhal et al. 2015), while the LC–ESI–MS requires growth of the microbial cells first and extraction of the compounds of interest before analysis. However, the LC–ESI–MS has been shown to be an enhanced method for the separation of different isoforms of the same analogues and homologues within a crude extract (in supernatant) produced using natural sources (Yang et al. 2015). Additionally, LC–ESI–MS is a powerful tool to utilise for quantitatively analysing complex compounds such as biosurfactants and can efficiently discriminate between different analogues and isoforms within a mixture of compounds.

Biosurfactant congeners display different physico–chemical properties in combination, which can differ from the physico-chemical properties observed in individual congeners (Bonmatin et al. 2003). A study conducted by Kracht et al. (1999) indicated that surfactin molecules (produced by *Bacillus subtilis* OKB 105) with 13 carbon atoms in their hydrophobic moiety exhibited low antiviral activity, while the surfactin isoform with 15 carbon atoms displayed the highest antiviral activity. In addition, the presence of a single negative charge also contributed to an increased antiviral activity. Studies have indicated that the microbial strains utilised for glycolipid or lipopeptide production have an influence on the yield and composition of the compounds synthesised, which in turn has an effect on their antimicrobial activity (Déziel et al. 1999; HoŠková et al. 2013).

The antimicrobial property of biosurfactants rely on different mechanisms to destroy target organisms as compared to conventional antibiotics (Banat et al. 2010) and they primarily destroy bacterial cells by directly disrupting the integrity of the plasma membrane or cell wall (Sang and Blecha 2008; Yount and Yeaman 2013). Most of the glycolipid and lipopeptide based biosurfactant compounds displaying antimicrobial properties, were extracted from microorganisms isolated from marine, terrestrial and sites contaminated by hydrocarbon based compounds (Abalos et al. 2001; Das et al. 2008; Sharma et al. 2014; 2015). Currently there is limited research on biosurfactant compounds produced by bacterial strains isolated from wastewater.

The current study focused on the purification and characterisation of antimicrobial glycolipid and lipopeptide biosurfactant compounds respectively, produced by *Pseudomonas aeruginosa* (*P. aeruginosa*) ST5 and *Bacillus amyloliquefaciens* (*B. amyloliquefaciens*) ST34 strains that were isolated from a local wastewater treatment plant. This aim was achieved by obtaining crude biosurfactant compounds from the *B. amyloliquefaciens* ST34 and *P. aeruginosa* ST5 strains grown on mineral salt medium (supplemented with glycerol) as well as nutrient agar, using acid-precipitation followed by a rapid solvent extraction method. An ESI–MS coupled with ultraperformance liquid chromatography (UPLC) method, denoted UPLC–MS, was developed for the characterisation of the biosurfactant extracts by using commercially available lipopeptides and glycolipids as standards. Finally, various opportunistic, pathogenic and antibiotic resistant bacteria and fungal strains were utilised for the assessment of the antimicrobial activity of the crude biosurfactant extracts obtained from the respective isolates.

## Materials and methods

### Bacterial isolates, media composition and biosurfactant production conditions

Biosurfactant producing bacteria were isolated from wastewater samples collected from Stellenbosch wastewater treatment plant in the Western Cape, South Africa (GPS co-ordinates: −33.943505, 18.824584) as described by Ndlovu et al. (2016). The bacterial isolates ST34, identified as *B. amyloliquefaciens* (collection number SARCC 696 at the South African Rhizobium Culture Collection) and ST5, identified as *P. aeruginosa* (collection number SARCC 697 at the South African Rhizobium Culture Collection), using molecular characterisation (Ndlovu et al. 2016), were utilised in the current study for biosurfactant production. Henceforth the *B. amyloliquefaciens* and *P. aeruginosa* isolates will be referred to by their code identifiers, ST34 and ST5, respectively. The bacterial cultures were maintained in 40% glycerol at −80 °C. An inoculum of the glycerol stock of ST34 and ST5 was streaked onto a nutrient agar (NA) plate which was incubated for 18–24 h at 37 °C. A single colony from each respective NA culture was then used to inoculate 5 mL sterile mineral salt medium (MSM) to prepare seed cultures. The MSM utilised for biosurfactant production was composed of the following: 0.1% KH_2_PO_4_, 0.1% K_2_HPO_4_, 0.02% MgSO_4_·7H_2_O, 0.002% CaCl_2_·2H_2_O, 0.005% FeCl_3_·6H_2_O and 0.2% NaNO_3_ and 3% glycerol as the main carbon and energy source, with the pH of the medium adjusted to 6.8 (Silva et al. 2010). The cultivation conditions for preparation of the seed culture were 30 °C, at 200 rpm with an incubation time of 18–24 h. After seed culture preparation, a 2% cell suspension of 0.7 optical density (OD) at 600 nm, which corresponded to approximately 10^7^ colony forming units (CFU) mL^−1^, was inoculated into 500 mL baffled flasks containing 100 mL MSM. The broth cultures were incubated on a 200 rpm orbital shaker (MRCLAB, London, UK) for 120 h at 30 °C.

### Extraction and partial purification of the biosurfactants

The crude biosurfactant compounds produced by ST34 and ST5 were obtained from the culture supernatant by a combination of acid and solvent extraction methods. Briefly, after 5 days of culturing the isolates in glycerol-MSM, the culture (100 mL) was centrifuged at 11,305×*g* for 30 min at 4 °C to remove microbial cells. The presence of surface active compounds in the supernatant was then verified using the oil spreading method as previously described by Ndlovu et al. (2016). Thereafter the supernatants were acidified to a pH of approximately 2 using hydrochloric acid (HCl, Merck, Darmstadt, Germany) as previously described by Das et al. (2008) and were stored overnight at 4 °C in order to precipitate the biosurfactant compounds. The precipitate was then harvested by centrifugation at 11,305×*g* for 30 min at 4 °C, and the pellet was washed with 50 mL of analytical quality water (prepared through a MilliQ system from Millipore, Billerica, USA), with the pH adjusted to 7.5 (Das et al. 2008). The respective insoluble fraction was then lyophilised and dissolved in 15% (*v/v*) methanol (Merck, Darmstadt, Germany) (crude extracts obtained from ST34 and ST5), transferred into analytically weighed sterile vials and lyophilised again. The extracts (ST34 and ST5) were analytically weighed and dissolved in 15% methanol to obtain a 1.00 mg mL^−1^ concentration, which was used for the characterisation and antimicrobial analysis (see list of test microbial strains in Tables 1 and 2). The methanol soluble fractions were lyophilised, further extracted using 70% acetonitrile and then lyophilised again. The extracts (ST34 and ST5) were analytically weighed and dissolved in 15% acetonitrile to obtain a 1.00 mg mL^−1^ concentration for analysis using the UPLC–MS.

**Table 1:** Antibacterial activity of the biosurfactant extracts (1.00 mg mL^−1^) against a panel of Gram-negative and Gram-positive bacterial isolates

Organism (strain number)	Source	Antibacterial inhibition zone diameter (mm) ± SD	
		Surfactin extract (0.26 ± 0.09 mg mL^−1^)	Rhamnolipid extract (1.12 ± 0.08 mg mL^−1^)
Gram-negative target organism			
*Escherichia coli* (ATCC 417373)	ATCC	13 ± 0	13.5 ± 0.4
*E. coli* (ATCC 13706)	ATCC	10 ± 0	29.3 ± 0.9
Enteroinvasive *E. coli* (ATCC 43892)	ATCC	15 ± 0	22.7 ± 2.1
^G^Enteropathogenic *E. coli* (B170)	ATCC	18.3 ± 0.5	20.3 ± 0.5
Enterohaemorhagic *E. coli* (O157:H7)	ATCC	13.7 ± 0.5	13.7 ± 0.5
Enterotoxigenic *E. coli* (H10407)	ATCC	17.7 ± 1.2	13 ± 0
Enteroaggregative *E. coli* (3591-87)	ATCC	12.3 ± 0.5	24.3 ± 1.2
*Klebsiella pneumoniae* (ATCC 10031)	ATCC	14 ± 1.6	13.5 ± 0.5
*Salmonella typhimurium (*ATCC 14028)	ATCC	25.3 ± 1.2	20.3 ± 0.5
*Serratia marcescens* (ATCC 13880)	ATCC	12.7 ± 0.9	14 ± 0
*K. pneumoniae* (P2)	Clinical	13 ± 0.8	11.7 ± 0.9
*K. pneumoniae* (P3)	Clinical	13.3 ± 0.2	8.3 ± 0.5
*Salmonella enterica* (SE19)	Environment	12.5 ± 0.5	14 ± 0
*Acinetobacter* sp. (F1S6)	Environment	12.3 ± 0.5	13 ± 1.4
*Serratia* sp. (SM14)	Environment	11.7 ± 0.9	14.3 ± 1.2
*Serratia* sp. (L8)	Environment	12.5 ± 0.5	9.8 ± 0.8
*Enterobacter* sp. (E11)	Environment	11.3 ± 0.5	13 ± 0.8
*Enterobacter* sp. (E22)	Environment	14.2 ± 0.6	13 ± 0.8
*E. coli* (K4CCA)	Environment	14.5 ± 0.5	17.7 ± 1.9
*K. pneumoniae* (k2a)	Environment	15.3 ± 0.5	13.7 ± 0.5
Gram-positive target organism			
^O^ *Staphylococcus aureus* (ATCC 25923)	ATCC	14.7 ± 0.5	13.7 ± 0.5
*B. cereus* (ATCC 10876)	ATCC	10.3 ± 0.5	13 ± 0.8
*B. cereus* (LMG 13569)	ATCC	13 ± 0.8	17 ± 1.4
*Enterococcus faecalis* (S1)	Clinical	18.7 ± 0.9	10.7 ± 0.5
*Enterococcus faecalis* (S2)	Clinical	18.3 ± 1.2	21.7 ± 2.4
^G,O,P,T^MRSA *(Xen 30)*	Clinical	15.3 ± 0.5	13.3 ± 0.5
*Bacillus cereus* (ST18)	Environment	Inactive	22.3 ± 0.9
*Enterococcus* sp. (C513)	Environment	12.3 ± 0.5	15.7 ± 0.5
*Micrococcus* sp. (AQ4S2)	Environment	14 ± 0	14 ± 1
*S. aureus* (C2)	Environment	11.5 ± 0.5	14 ± 0
*S. aureus* (C3)	Environment	12 ± 0	11 ± 0

The surfactin and rhamnolipid extracts were observed to be at 32.8 and 34.4% purity, respectively

Values are the means ± standard deviations (SD) of triplicate measurements; ATCC American Type Culture, ^O^ resistant to Oxacillin, ^G^ resistant to Gentamicin, ^T^ resistant to Tetracycline, ^P^ resistant to Penicillin G

**Table 2:** In vitro antifungal activity of the surfactin and rhamnolipid biosurfactant extracts (1.00 mg mL^−1^) against a panel of clinical and environmental fungal isolates as determined by agar disc diffusion method

Organism	Antifungal zone diameter (mm)	
	Surfactin extract (0.26 ± 0.09 mg mL^−1^)	Rhamnolipid extract (1.12 ± 0.08 mg mL^−1^)
^a^ *Cryptococcus neoformans* CAB1063	Inactive	13 ± 0.8
^a^ *Cryptococcus neoformans* CAB1067	11.7 ± 0.5	14.3 ± 3.3
^a^ *Cryptococcus neoformans* CAB1055	15.3 ± 0.5	11.3 ± 0.9
^a^ *Candida albicans* 8911	13.3 ± 0.5	14.7 ± 0.5
^a^ *Candida albicans* 8912	13.3 ± 0.5	11.7 ± 0.5
^b^ *Cryptococcus neoformans* CAB1034	Inactive	18.3 ± 0.8
^b^ *Cryptococcus neoformans* CAB831	11.7 ± 1.7	15.3 ± 1.9
^b^ *Cryptococcus neoformans* CAB842	12.3 ± 0.9	Inactive
^b^ *Cryptococcus neoformans* CAB844	15.3 ± 1.2	16.7 ± 1.7
^b^ *Candida albicans 1085*	Inactive	14 ± 0.8

The surfactin and rhamnolipid extracts were observed to be at 32.8 and 34.4% purity, respectively

^a^ Clinical strain

^b^ Environmental strain

The ST34 and ST5 isolates were also cultured in duplicate on NA plates and NA slants (10 mL test tube) for approximately 5 days at 30 °C. Five millilitres of 70% acetonitrile (Romil, Cambridge, UK) was added to the NA plate cultures, which were then placed on a Bio dancer shaker (New Brunswick Scientific, Enfield, USA) at a speed of 5 rpm for approximately 5 min. The acetonitrile mixture was decanted into a sterile McCartney bottle. For the NA slant cultures, 5 mL of 70% acetonitrile was added to the test tube, the culture was vortexed for approximately 2 min, where after the acetonitrile mixture was decanted into a sterile McCartney bottle. The lyophilised acetonitrile extracts obtained from NA plates and slants were then suspended in 1 mL sterile analytical quality water, the soluble supernatant was removed and the insoluble fractions were lyophilised and weighed analytically. After weighing, the extracts were dissolved in 15% acetonitrile to obtain a 1.00 mg mL^−1^ concentration, which was used for the characterisation of the biosurfactants produced by each bacterial strain.

### Analysis with ultra-performance liquid chromatography linked to electrospray ionisation mass spectrometry

Mass spectrometry analyses were performed in the LCMS Central Analytical Facility at the Stellenbosch University. A Waters Quadrupole Time-of-Flight Synapt G2 (Waters Corporation, Miliford, USA) mass spectrometer was utilised for the ESI–MS and was coupled to an Acquity UPLC for the UPLC–MS analysis of the biosurfactant extracts. Three microlitres of the standards and acetonitrile soluble extracts (glycerol-MSM) obtained from ST34 and ST5 at 1.00 mg mL^−1^ were directly injected into a Z spray electrospray ionisation source for direct mass analysis. The identities of the biosurfactant compounds were confirmed with high resolution MS by comparing it with the mass/charge ratio (*m/z*) obtained for bacillomycin, fengycin and mycosubtilin (LipoFabrik, Lille, France) and iturin A, surfactin and rhamnolipid (Sigma-Aldrich, St. Louis, USA) as standards.

For UPLC–MS analysis 3 µL of each standard, extracts obtained from glycerol-MSM liquid culture, NA surface culture in a petri-dish and NA slant cultures in test tubes was injected and separated on an UPLC C18 reverse-phase analytical column (Acquity UPLC^®^ HSS T3, 1.8 µm particle size, 2.1 × 150 mm, Waters corporation, Dublin, Ireland) at a flow rate of 0.300 mL min^−1^ using a 0.1% formic acid (A) to acetonitrile (B) gradient (60% A from 0 to 0.5 min for loading, gradient was from 40 to 95% B from 0.5 to 11 min and then 95 to 40% B from 15 to 18 min). The UPLC–MS profiles of the biosurfactant compounds were compared to those obtained for bacillomycin, fengycin, iturin A, surfactin, rhamnolipid and mycosubtilin standards. Moreover, the concentration of surfactin and rhamnolipid in the extracts obtained from *B.* *amyloliquefaciens* ST34 and *P. aeruginosa* ST5 strains were analysed using a UPLC–MS method described by Ndlovu (2017).

For both direct ESI–MS and UPLC–MS analyses, the analytes were subjected to a capillary voltage of 3 kV, cone voltage of 15 V and a source temperature of 120 °C. Data acquisition in the positive mode was performed by MS scanning a second analyser through the *m/z* range of 200–3000 Da and the data was thereafter analysed using Masslynx software version 4.1 (Waters Corporation, Milford, USA).

### Determination of antimicrobial activity: agar disc susceptibility test

The antimicrobial activity of the extracts obtained from ST34 and ST5, was analysed against various actively growing target reference strains [from American Type Culture Collection (ATCC)], environmental and clinical Gram-positive and Gram-negative microbial strains (Table 1) as well as fungal strains (Table 2) on Mueller Hinton agar (MHA) (Merck, Darmstadt, Germany). The bacterial environmental strains were isolated by our research group from rainwater tanks and surface water (Plankenburg River, Stellenbosch, South Africa), while the clinical strains were obtained from laboratories in the Department of Microbiology at Stellenbosch University (Stellenbosch, South Africa). Fungal strains isolated from surface water (Benadé et al. 2016) and clinical samples obtained from the Environmental Biotechnology laboratory in the Department of Microbiology (Stellenbosch University, South Africa) were also included as antimicrobial test strains against ST34 and ST5 extracts. Briefly, the crude biosurfactant extracts were dissolved in 15% (*v/v*) methanol (70% acetonitrile was also utilised for the antimicrobial assays; results were however comparable or lower than the results obtained for the crude extract) and were filtered through a 0.22 µm low protein binding non-pyrogenic syringe filter (Pall Life Sciences, Ann Arbor, USA). A 100 µL overnight culture of the test microbial isolates (Tables 1, 2), which had been grown in Luria–Bertani broth (Merck, Darmstadt, Germany), was then spread plated onto the MHA to create a microbial lawn. Thereafter, using sterile tweezers, 6 mm filter paper discs (Oxoid, Basingstoke, UK) were placed onto the lawn and 50 µL of the biosurfactant extract (1.00 mg mL^−1^), obtained from either ST34 or ST5, was pipetted directly onto the filter paper in order to create an antimicrobial disc. The antimicrobial tests were performed with a negative control (MHA plus test bacterial strain) and three positive controls [MHA plus pure surfactin and rhamnolipid purchased from Sigma, USA, against the representative Gram-positive *Staphylococcus aureus* ATCC 25,923, the representative Gram-negative *Escherichia coli* ATCC 13,706 and the fungal isolate *Cryptococcus neoformans* CAB1055]. All tests were performed in triplicate. All the MHA plates were then incubated at 37 °C for 24–48 h where after the diameter of the zone of inhibition around the inoculated paper disc was measured (Das et al. 2008).

### Statistical analysis

The diameters of the zones of inhibition produced by the ST34 and ST5 extracts against various microbial strains analysed in the current study, were expressed as mean values ± standard deviation. The student’s *t* test was then utilised to determine the statistical significant difference between the diameters of the zones of inhibition between the extracts produced by ST34 and ST5, respectively, against the test bacterial and fungal strains. The P values of less than 0.05 (p < 0.05) were considered significant.

## Results

### Direct ESI–MS analysis for solvent extracted biosurfactant compounds produced by ST34

**Fig. 1:**
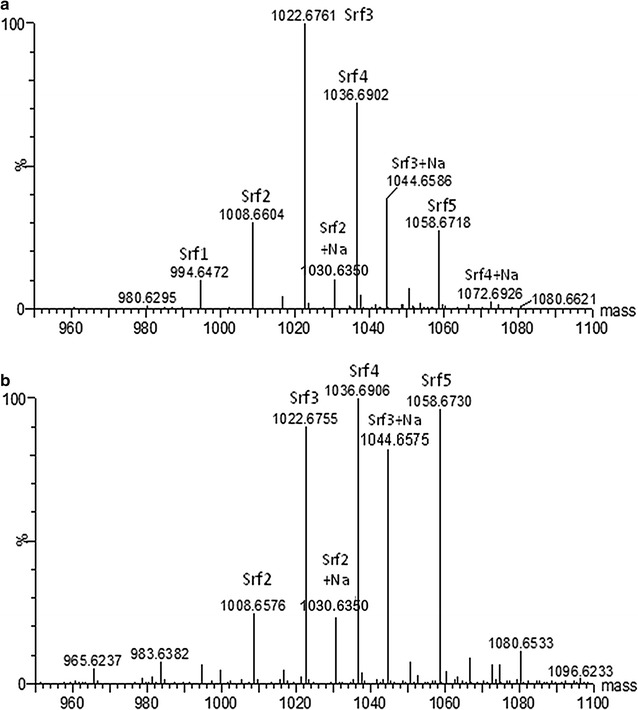
ESI–MS analysis of the ST34 glycerol-MSM extract (**a**) and surfactin standard (**b**). The positive mass spectrum generated with MaxEnt 3 is shown. The indicated masses are [*M*_r_+H] = *m/z* values of singly charged species. Refer to Table 3 for identities of Srf1-5 and expected *m/z* and *M*_r_ values

Solvent extracts of the glycerol-MSM liquid culture obtained from ST34 were subjected to direct infusion using positive mode ESI–MS in order to determine the accurate molecular mass (compound identity) for the solvent extracted biosurfactant compounds. The spectra of the possible biosurfactant compounds produced by ST34 were compared to the surfactin, mycosubtilin, bacitracin, iturin A and fengycin standards. However, the compounds detected only corresponded to the profile observed for the surfactin standard, hence only the results for surfactin standard are depicted in Fig. 1. In the ESI–MS spectrum of the ST34 extract from glycerol-MSM, a cluster of *m/z* peaks with a difference of approximately 14 or 22 or 28 atomic mass units (amu) in their molecular ion species were detected, revealing five groups of analogue molecules (Fig. 1). The spectra in positive mode showed the main groups of molecular ions at *m/z* 994.65, 1008.66, 1022.68, and 1036.69 which corresponded to the protonated singly charged species [M+ H]^+^ (Fig. 1; Table 3). Their corresponding sodium adducts [M+Na]^+^ were also detected at *m/z* 1016.63, 1030.64, 1044.65 and 1058.66 (Fig. 1a; Table 3). For the standard surfactin, the spectra in the positive mode displayed the main groups of molecular ions at *m/z* 1008.66, 1022.68 and 1036.66 which corresponded to the protonated singly charged species [M+H]^+^ (Fig. 1c; Table 3). Their sodium adducts [M+Na]^+^ were also detected at *m/z* 1044.66 and 1058.68.

**Table 3:** Summary of the detected surfactin lipopeptides extracted from cultures of *B. amyloliquefaciens* ST34, as detected using high resolution mass spectrometry (<10 ppm)

Surfactin group (Abbr)	UPLC Rt (min)^a^	Characterised and proposed* peptide sequences in surfactin group	Mono-isotopic Exp/Theor *M*_r_	Protonated specie Exp/Theor *m/z*	Sodiated specie Exp/Theor *m/z*
Surfactin 1 (Srf1)	10.6; 11.2	Cyclo[(**C**_**13**_ **H**_**24**_ **O**_**2**_)-L-Glu-**L-Leu**-D-Leu-L-Val-L-Asp-L-Leu-**L-Val**] Cyclo[(**C**_**13**_ **H**_**24**_ **O**_**2**_)-L-Glu-**L-Ile**-D-Leu-L-Val-L-Asp-L-Leu-**L-Val**]	993.6376 993.6403	994.6472 994.6481	1016.6265 1016.6190
Surfactin 2 (Srf2)	11.0; 11.2; 11.9	Cyclo[(**C**_**14**_ **H**_**26**_ **O**_**2**_)-L-Glu-**L-Leu**-D-Leu-L-Val-L-Asp-L-Leu-**L-Val**] Cyclo[(**C**_**14**_ **H**_**26**_ **O**_**2**_)-L-Glu-**L-Ile**-D-Leu-L-Val-L-Asp-L-Leu-**L-Val**]	1007.6521 1007.6552	1008.6604 1008.6596	1030.6350 1030.6328
		Cyclo-[(**C**_**13**_ **H**_**24**_ **O**_**2**_)-L-Glu-**L-Leu**-D-Leu-L-Val-L-Asp-L-Leu-**L-Leu**] Cyclo[(**C**_**13**_ **H**_**24**_ **O**_**2**_)-L-Glu-**L-Leu**-D-Leu-L-Val-L-Asp-L-Leu-**L-Ile**] *Cyclo-[(**C**_**13**_ **H**_**24**_ **O**_2_)-L-Glu-**L-Ile**-D-Leu-L-Val-L-Asp-L-Leu-**L-Leu**] *Cyclo-[(**C**_**13**_ **H**_**24**_ **O**_**2**_)-L-Glu-**L-Ile**-D-Leu-L-Val-L-Asp-L-Leu-**L-Ile**]			
Surfactin 3 (Srf3)	11.6; 11.7; 12.3	Cyclo[(**C**_**15**_ **H**_**28**_ **O**_**2**_)-L-Glu-**L-Leu**-D-Leu-L-Val-L-Asp-L-Leu-**L-Val**] Cyclo[(**C**_**15**_ **H**_**28**_ **O**_**2**_)-L-Glu-**L-Ile**-D-Leu-L-Val-L-Asp-L-Leu-**L-Val**]	1021.6693 1021.6715	1022.6780 1022.6752	1044.6586 1044.6494
		Cyclo[(**C**_**14**_ **H**_**26**_ **O**_**2**_)-L-Glu-**L-Leu**-D-Leu-L-Val-L-Asp-L-Leu-**L-Leu**] Cyclo[(**C**_**14**_ **H**_**26**_ **O**_**2**_)-L-Glu-**L-Leu**-D-Leu-L-Val-L-Asp-L-Leu-**L-Ile**] *Cyclo-[(**C**_**14**_ **H**_**26**_ **O**_**2**_)-L-Glu-**L-Ile**-D-Leu-L-Val-L-Asp-L-Leu-**L-Leu**] *Cyclo-[(**C**_**14**_ **H**_**26**_ **O**_**2**_)-L-Glu-**L-Ile**-D-Leu-L-Val-L-Asp-L-Leu-**L-Ile**]			
Surfactin 4 (Srf4)	12.1; 12.2	Cyclo[(**C**_**15**_ **H**_**28**_ **O**_**2**_)-L-Glu-**L-Leu**-D-Leu-L-Val-L-Asp-L-Leu-**L-Leu**] Cyclo[(**C**_**15**_ **H**_**28**_ **O**_**2**_)-L-Glu-**L-Leu**-D-Leu-L-Val-L-Asp-L-Leu-**L-Ile**] *Cyclo[(**C**_**15**_ **H**_**28**_ **O**_**2**_)-L-Glu-**L-Ile**-D-Leu-L-Val-L-Asp-L-Leu-**L-Leu**] Cyclo[(**C**_**15**_ **H**_**28**_ **O**_**2**_)-L-Glu-**L-Ile**-D-Leu-L-Val-L-Asp-L-Leu-**L-Ile**]	1035.6819 1035.6881	1036.6902 1036.6909	1058.6718 1058.6662
Surfactin 5 (Srf5)	12.6; 12.7	Cyclo[(**C**_**16**_ **H**_**30**_ **O**_**2**_)-L-Glu-**L-Leu**-D-Leu-L-Val-L-Asp-L-Leu-**L-Leu**] *Cyclo[(**C**_**16**_ **H**_**30**_ **O**_**2**_)-L-Glu-**L-Leu**-D-Leu-L-Val-L-Asp-L-Leu-**L-Ile**] *Cyclo[(**C**_**16**_ **H**_**30**_ **O**_**2**_)-L-Glu-**L-Ile**-D-Leu-L-Val-L-Asp-L-Leu-**L-Leu**] *Cyclo[(**C**_**16**_ **H**_**30**_ **O**_**2**_)-L-Glu-**L-Ile**-D-Leu-L-Val-L-Asp-L-Leu-**L-Ile**]	1049.6992 1049.7032	1050.7120 1050.7066	1072.6926 1072.6886

Their proposed chemical structures, theoretical (Theor) and experimental (Exp) *M*_r_ and monoisotopic *m/z* values, as well as observed UPLC retention times for representative examples are provided

^a^ UPLC retention time of main peaks corresponding to the group’s *m/z* value

The singly charged protonated molecular species [M+H]^+^ at *m/z* 994.65, 1008.66, 1022.68 and 1036.66 and their corresponding singly charged sodiated molecules [M+Na]^+^ (1016.6, 1030.6, 1044.66 and 1058.68) all differed by 14 or 28 amu (Table 3). The detected high resolution *M*_r_ values (ppm < 10) of the possible surfactin analogues in the ST34 extract corresponded to that of the C_13_, C_14_, C_15_ and C_16_ surfactin analogues (Srf1-5) in a standard surfactin, confirming their identity (Fig. 1; Table 3).

### ESI–MS and UPLC–MS analysis of solvent extracted biosurfactant compounds produced by ST34

**Fig. 2:**
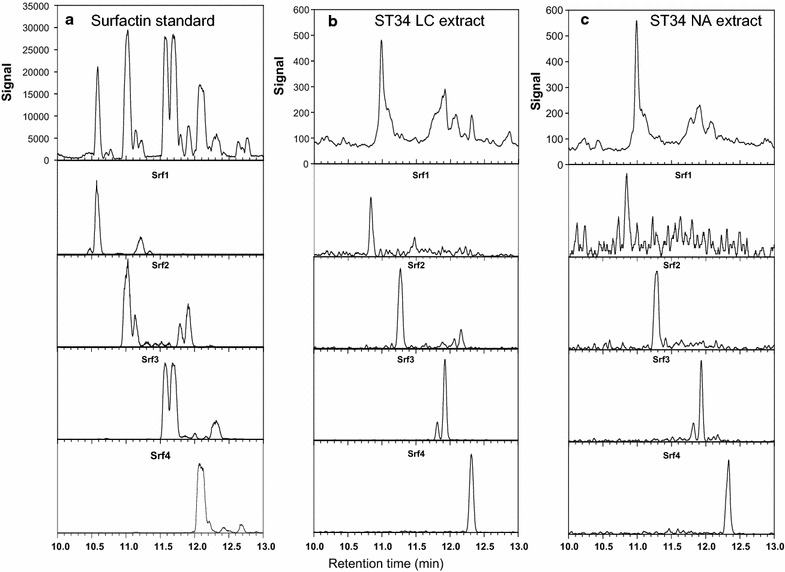
UPLC–MS profiles of surfactin standard (**a**), ST34 glycerol-MSM liquid culture (ST34LC) extract (**b**) and ST34 nutrient agar surface culture (ST34NA) (**c**) showing the five major surfactin groups. The *top row* profiles show the signal of positive molecular ions detected between 10 and 13 min. Note the difference in* Y axis* which are a direct indication of amounts. The profiles below each *top row* spectrum show the extracted spectra of the five surfactin groups with Srf1 = *m/z* 994.6, Srf2 = m/z 1008.7; Srf3 = *m/z* 1022.7, Srf4 = *m/z* 1035.7 and Srf5 = *m/z* 1050.7

An optimised UPLC–MS method was employed to analyse the lipopeptide biosurfactant extract obtained from ST34 cultured in glycerol-MSM (ST34LC) is shown in Fig. 2b (compared with the surfactin standard; Fig. 2a). The UPLC–MS profiles of the biosurfactant compounds produced by ST34 corresponded very well with the profile observed for the surfactin standard (Fig. 2a). Surface culture on NA (ST34NA) in test tubes (ST34NA-TSC) and petri dishes (ST34NA-PDC) were also utilised to produce biosurfactants by ST34, in order to increase the probability of detecting lipopeptides in/on different growth media. As the NA cultures were extracted with 70% acetonitrile in water (*v/v*), the ST34LC (original crude extract) was further extracted with 70% acetonitrile (ST34LC-AE) and analysed. The comparative UPLC–MS profiles of the extracts are shown in Fig. 2. The UPLC–MS profiles of the surfactin standard and the extracts produced by ST34 showed significant peaks at retention times between 10 and 13 min.

From basic reverse-phase chromatography principles, it is expected that the surfactin species with the longer fatty acyl chains will elute at a later retention time (R_t_) from the C_18_ matrix. This was indeed the case, with the sequence of surfactin groups eluted as follows, surfactin 1 (Srf1) (R_t_ 10.6; 11.2 min), Srf2 (R_t_ 11.0, 11.2, 11.9 min), Srf3 (R_t_ 11.6, 11.7, 12.3 min), Srf4 (R_t_ 12.1, 12.2 min) and Srf5 (R_t_ 12.6, 12.7 min) (Fig. 2; Table 3). In the surfactin groups, Ile/Leu analogues will elute closer or together, while the slightly smaller and less hydrophobic Val analogues will elute earlier. It should be noted that the peptide identities within specific surfactin groups were not fully explored as it was beyond the scope of the study. However, this UPLC–MS methodology has the potential to be extended to include tandem mass spectrometry and ion mobility on the Synapt G2 in future studies.

For the glycerol-MSM culture extracts, five peaks/peak clusters were observed on the UPLC–MS profile which corresponded to five surfactin groups. The five surfactin groups (Srf1, Srf2, Srf3, Srf4 and Srf5) exhibited similar retention times as the surfactin standard (Fig. 2b). As indicated, the ST34 was also cultivated in NA in order to increase the probability of detecting the produced biosurfactant compounds. The extracted UPLC–MS profiles for the NA extracts showed major peaks which corresponded to Srf2, Srf3 and Srf4, while traces of Srf1 and Srf5 surfactin analogues were also detected (Fig. 2c).

**Fig. 3:**
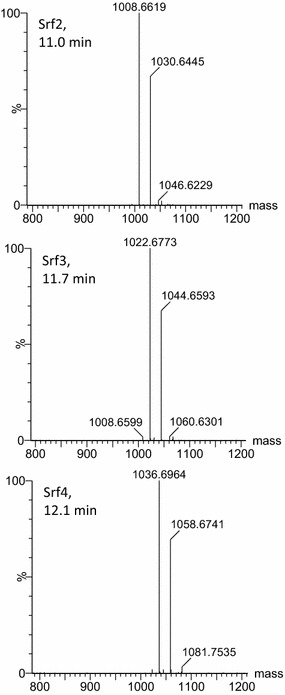
Examples of the ESI–MS mass spectra of three major surfactin groups detected with UPLC–MS. The positive mass spectrum generated with MaxEnt 3 is shown. The indicated masses are [*M*_r_+H] = *m/z* values of singly charged species. Refer to Table 3 for identities of Srf1-5 and expected *m/z* and *M*_r_ values

A detailed analysis of some of the major peaks in the UPLC–MS profiles of the ST34LC extract (glycerol-MSM culture extract) revealed that these peaks contained both the protonated molecular species, as well as the sodiated species of the surfactin group (Fig. 3). The ST34LC extract produced two major peaks at 11.0 and 11.7 min. The peak at 11.0 min corresponded to the lipopeptides in the Srf3 group which yielded a surfactin analogue with *M*_r_ of 1021.67 (expected *M*_r_ of 1021.67) and its sodium adduct at 1044.65 (expected *M*_r_ of 1044.65) (Fig. 3; Table 3). It was also observed that next to the main peak (11.0 min, Fig. 2b) obtained in the ST34 glycerol extract (ST34LC), were two peaks at 11.2 and 11.9 min that corresponded to the Srf2 (Fig. 2b; Table 3). The peaks at 11.2 and 11.9 min both corresponded to the surfactin analogues with *M*_r_ of 1007.65, which existed with their sodium adducts with *M*_r_ of 1030.64. The other major peak for the ST34LC extract was observed at 11.7 min and corresponded to the Srf3 group that showed an analogue with *M*_r_ of 1021.67 (expected *M*_r_ of 1021.67) and its sodium adduct at 1044.65 (expected *M*_r_ of 1044.65) (Fig. 3; Table 3). A detailed analysis of the major peak observed for the ST34NA extract (NA = petri dish and test tube slant cultures; Fig. 2b) was then observed at 12.1 min. The peak corresponded to the Srf4 group which yielded a surfactin analogue with *M*_r_ of 1035.68 (expected *M*_*r*_ of 1035.69) (Fig. 3; Table 3).

From the accurate *M*_r_ values and corresponding UPLC profiles it was then concluded that the ST34 extract contained all five surfactin groups (Fig. 3; Table 3). After further comparison with all the commercial standards utilised in the current study (not shown), results showed that surfactin was the only lipopeptide biosurfactant detected in the ST34 extracts obtained from the glycerol-MSM and NA using the production conditions (glycerol-MSM, temperature and the agitation speed) applied in the current study.

The relative abundance of each surfactin group within the complex surfactin lipopeptides in the ST34 extract obtained from the glycerol-MSM and NA cultures was inferred from the *M*_*r*_ extracted chromatograms by combining the peak areas of each surfactin group eluting between 10 and 13 min. The relative content for each surfactin group is illustrated in Additional file 1: Figure S1, and it showed that the Srf1 and Srf5 groups were below 5% relative abundance in the ST34 extracts obtained from the glycerol-MSM and the NA media (both the test tube slant and petri dish cultures). The Srf2, Srf3 and Srf4 were the main surfactin groups detected in the ST34 extracts illustrated in Additional file 1: Figure S1. The NA test tube slant culture produced the Srf4 group in higher concentrations, with a relative abundance of approximately 60% (Additional file 1: Figure S1). In contrast, the glycerol-MSM liquid cultures produced the Srf3 in higher concentrations, with a relative abundance of approximately 43% (Additional file 1: Figure S1).

### Direct ESI–MS analysis of solvent extracted biosurfactant compounds produced by ST5

**Fig. 4:**
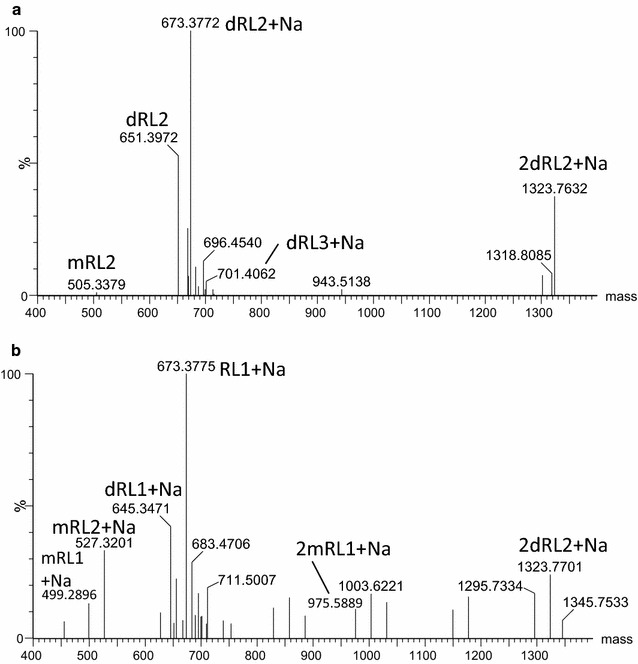
ESI–MS analysis of the ST5 glycerol-MSM extract (**a**) and rhamnolipid standard (**b**). The positive mass spectrum generated with MaxEnt 3 is shown. The indicated masses are [*M*_r_+H] = *m/z* values of singly charged species. Refer to Table 4 for identities of RL 1-4 and expected *m/z* and *M*_r_ values

Solvent extracts of the glycerol-MSM liquid culture obtained from ST5 were subjected to direct infusion using the positive ESI–MS in order to determine the accurate molecular mass (compound identity) for the solvent extracted biosurfactant compounds. The spectra of the possible biosurfactant compounds produced were compared to the rhamnolipid, surfactin, mycosubtilin, bacitracin, iturin A and fengycin standards. However, the compounds detected only corresponded to the profile observed for the rhamnolipid standard, hence only the results for rhamnolipid standard are depicted in Fig. 4. In the positive mode ESI–MS for the ST5 extract obtained from the glycerol-MSM ST5 culture we observed a series of sodiated singly charged ions at *m/z* values of 673.38, 645.35, 527.32 and 499.29 (Fig. 4, Table 4). Corresponding sodiated dimers [2 M−H+Na]^+^ at *m/z*, 1323.77, and 975.59 (Fig. 4) were also generally detected. For the standard rhamnolipid, the spectra in positive mode showed major molecular ions at *m/z* 651.40, 673.38 and 1323.77, which corresponded to the singly charged species, [M+H]^+^ and [M+Na]^+^, as well as sodiated dimer (Fig. 4). While analysing the full ion spectrum of the rhamnolipid standard, a series of ions of *m/z* values corresponding to the fragment or molecular ions of the 3-(3-hydroxyalkanoyloxy) alkanoic acids (HAAs) were also observed (results not shown). These HAAs were also detected with the rhamnolipid congeners with *m*/*z* values of 331.2, 359.3 and 387.3, which correspond to protonated [M+H]^+^ molecular ions of a HAA containing one 3-hydroxydecanoate (C_10_) and one 3-hydroxyoctanoate (C_8_) moiety, two C_10_ moieties and one C_10_ and one 3-hydroxydodecanoate (C_12_) moiety, respectively, were the most abundant (refer to discussion below and Fig. 6d).

**Table 4:** Summary of the rhamnolipids extracted from cultures of *P. aeruginosa* ST5, as detected with high resolution mass spectrometry (<10 ppm)

Rhamnolipid group (Abbr)	UPLC Rt (min)^a^	Proposed structures of rhamnolipids	Mono-isotopic Exp/Theor *M*_r_	Protonated specie Exp/Theor *m/z*	Sodiated specie Exp/Theor *m/z*	Sodiated dimeric specie Exp/Theor *m/z*
mRL1	7.23	Rha–C_8_–C_10_ Rha–C_10_–C_8_	476.3047 476.2985	477.3089 477.3063	499.2896 499.2883	975.5889 975.5868
dRL1	6.32 6.45	Rha–Rha–C_8_–C_10_ Rha–Rha–C_10_–C_8_	622.3576 622.3564	623.3654 623.3642	645.3471 645.3462	1267.7074 1267.7026
mRL2	8.77 8.84	Rha–C_10_–C_10_	504.3305 504.3298	505.3383 505.3376	527.3201 527.3196	1031.6501 1031.6494
dRL2	7.84 7.97	Rha–Rha–C_10_–C_10_	650.3894 650.3877	651.3972 651.3955	673.3772 673.3775	1323.7701 1323.7652
mRL3	10.32	Rha–C_12_–C_10_ Rha–C_10_–C_12_	532.3640 532.3611	533.3700 533.3689	555.3546 555.3509	1087.7201 1087.7120
dRL3	9.40 9.46	Rha–Rha–C_12_–C_10_ Rha–Rha–C_10_–C_12_	678.4177 678.4190	679.4285 679.4268	701.4114 701.4088	1379.8352 1379.8278

Their proposed chemical structures, theoretical (Theor) and experimental (Exp) *M*_r_ and monoisotopic *m/z* values, as well as observed UPLC retention times for representative examples are provided

^a^ UPLC retention time of main peaks corresponding to the group’s *m/z* value

**Fig. 5:**
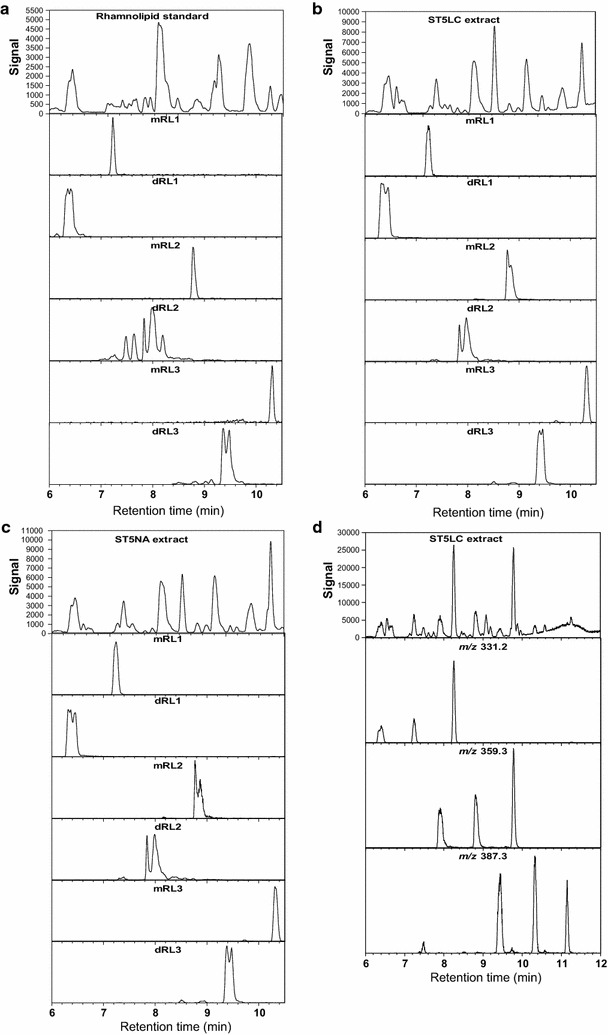
UPLC–MS profiles of rhamnolipid standard (**a**), ST5 glycerol-MSM liquid culture (ST5LC) extract (**b**) and ST5 nutrient agar surface culture (ST5NA) (**c**) showing the four major rhamnolipid groups. The *top row* profiles show the signal of positive molecular ions detected between 6 and 10 min. The profiles below each *top row* spectrum show the extracted spectra of the five rhamnolipid groups with RL1 = *m/z* 673.4, RL2 = m/z 645.3; RL3 = *m/z* 527.3 and RL4 = *m/z* 499.3. Profiles in **d** show the three types of HHAs (*m/z* 331.2, 359.3 and 387.3), either as precursors (third eluting peak) or fragments (first two eluting peaks) found in the ST5LC extract

The molecular mass of the possible rhamnolipid congeners detected in the ST5 extract were then determined from the molecular ions observed (Fig. 4; Table 4). The ST5 extract showed singly charged sodiated molecular species [M+Na]^+^ at *m/z* 645.35, 673.38, 701.41, 499.29, 527.32, 555.35 (Fig. 4), which is in agreement with *M*_*r*_ of the dirhamnolipids Rha–Rha–C_8_–C_10_/Rha–Rha–C_10_–C_8_ (dRL1), Rha–Rha–C_10_–C_10_ (dRL2), and Rha–Rha–C_12_–C_10_/Rha–Rha–C_10_–C_12_ and monorhamnolipids, Rha–C_8_–C_10_/Rha–C_10_–C_8_ (mRL1), Rha–C_10_–C_10_ (mRL2) and Rha–C_10_–C_12_/Rha–C_12_–C_10_ (mRL3), respectively (Table 4). Moreover, the *m*/*z* values at 331.25, 359.28 and 387.32, which corresponded to protonated [M+H]^+^ molecular ions of a HAA containing C_10_–C_8_/C_8_–C_10_, C_10_–C_10_ and C_10_–C_12_/C_12_–C_10_ moieties, respectively were detected in the ST5 extract (refer to discussion below and Fig. 5).

### ESI–MS and UPLC–MS analysis of solvent extracted biosurfactant compounds produced by ST5

As the chromatographic separation in UPLC–MS analyses limits the interference of counter ions, it is more likely to detect more rhamnolipid species in both the rhamnolipid standard and ST5 culture extracts, as well as quantify these compounds. Our UPLC–MS method was therefore also used to analyse the glycolipid biosurfactant extract obtained from ST5 cultured in glycerol-MSM (ST5LC) (Fig. 4b). Surface cultures on NA in test tubes were also utilised to produce biosurfactants by ST5, in order to increase the probability of detecting glycolipids on different media (Fig. 4c). The chromatographic profiles of the possible biosurfactant compounds produced were compared to the standards and analysis of results revealed that the profile obtained for ST5 only corresponded with the profile of the rhamnolipid standard (Fig. 4a). The comparative UPLC–MS profiles of the rhamnolipid standard and the extracts produced by ST5 exhibited significant peaks at retention times between 6 and 10.5 min. From basic reverse-phase chromatography principles, it is expected that the rhamnolipid species composed of two rhamnose and shorter HAA chain(s) will be eluted first, while the rhamnolipid with one rhamnose sugar and longer HAA chain(s) will elute later from the C_18_ matrix. This principle was observed for the rhamnolipid compounds produced by the ST5 strain, with the dirhamnolipids in the groups (dRL1, dRL2 and dRL3) eluting first and monorhamnolipid (mRL1, mRL2 and mRL3) eluting second in each group (Fig. 4a, b; Table 4).

For the glycerol-MSM culture extract, six peaks/peak clusters were observed in the UPLC–MS profile which corresponded to six rhamnolipid groups. The six rhamnolipid groups from ST5 liquid culture extracts yielded identical retention times and *m/z* values (Fig. 4b) to those of the rhamnolipid standard (Fig. 4a). The ST5 extract obtained from the NA in a test tube (not shown) also displayed the same major peaks which corresponded to dirhamnolipids (dRL1, dRL2 and dRL3) and their monorhamnolipids (mRL1, mRL2 and mRL3) (Table 4).

**Fig. 6:**
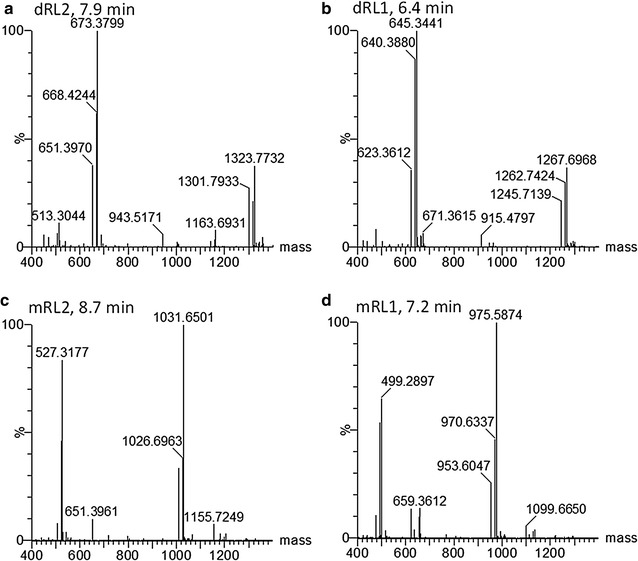
Examples of the ESI–MS mass spectra of major rhamnolipid groups detected with UPLC–MS (dRL1-2 and mRL1-2). Mass spectra were generated with MaxEnt 3. Refer to Table 4 for identities of dRL1-2 and mRL1-2 and expected *m/z* and *M*_r_ values

A more detailed analysis of some of the major peaks in the UPLC–MS profiles revealed that these peaks contained the free rhamnolipid congener, protonated and sodiated molecular species (Fig. 6). For example, the peak at 7.9 min corresponded to one of the glycolipid dRL2 group that showed a rhamnolipid congener with *M*_r_ of 650.39 (expected *M*_r_ of 650.39), the protonated ion at 651.40 (expected *m/z* of 651.40) and its sodium adduct at *m/z* 673.38 (expected *m/z* of 673.38) (Fig. 6a). The peak at 8.7 min corresponded to the corresponding mRL2 rhamnolipid congener with a *M*_r_ of 504.33 (expected *M*_r_ of 504.33), with its protonated species at *m/z* of 505.34 (expected *m/z* of 505.34 Da) and its sodium adduct at *m/z* 527.32 (expected *m/z* of 527.32) (Fig. 6b). The spectra for the monorhamnolipid mRL1 and its dirhamnolipid dRl1 is shown in Fig. 6c and d. Furthermore, the protonated and sodiated HAA fragments of C_10_–C_8_/C_8_–C_10,_ were also detected in the rhamnolipid mRL1 and dRL1 peaks (*m/z* 331.2 and 353.2) and HAA fragments of C_10_–C_10_ in the mRL2 and dRL2 congener peaks (*m/z* 359.3 and 381.3). Refer to Fig. 5d for the UPLC–MS profiles showing the detection of these major HAAs with *m*/*z* values of 331.2 and 359.3 in ST5LC extract.

The peak at 10.3 min corresponded to the mRL3 monorhamnolipid congener with a protonated molecular species at *M*_*r*_ 532.36 (expected *M*_*r*_ of 532.36), with its sodium adduct at *m/z* of 555.35 (expected 555.35). A dirhamnolipid Rha–Rha–C_10_–C_12_ or Rha–Rha–C_12_–C_10_ was also produced and was observed at R_t_ 9.4 min at *m/z* 701.41 (expected 701.41) (Fig. 5; Table 4). This identity of the lipid moiety was confirmed by the detection of the hydroxyl fragment of C_10_–C_12_/C_12_–C_10_ at *m/z* of 387.31 in the RL3 congeners. Refer to Fig. 5d for the UPLC–MS profiles showing the detection of the C_10_–C_12_/C_12_–C_10_ HAA ions with *m*/*z* at 387.3 in ST5LC extracts and the rhamnolipid standard.

Dimers of the sodiated [2 M−H+Na]^+^ dirhamnolipid Rha–Rha–C_10_–C_10_ (dRL2) and monorhamnolipid Rha–C_10_–C_10_ (mRL2) were observed at *m/z* 1323.77 and 1031.65, respectively (Table 4). Dimers of the sodiated [2 M−H+Na]^+^ dirhamnolipid Rha–Rha–C_10_–C_8_/Rha–Rha–C_8_–C_10_ (dRL1) and monorhamnolipid Rha–C_10_–C_8_/Rha–C_8_–C_10_ (mRL1) were observed at *m/z* 1267.71 and 975.59, respectively. Similarly, the sodiated dimers of the RL3 group was also detected (Table 4).

The glycerol-MSM and NA cultures of ST5 lead to the production of similar rhamnolipid profiles (Fig. 5). A total of six rhamnolipid groups (mRL1-3 and dRL1-3) were identified in both the rhamnolipid standard and ST5 culture extracts with high resolution ESI–MS (ppm < 10) and their proposed structures are presented in Table 4.

### Antimicrobial activity of biosurfactant extracts

The antimicrobial activity of the identified surfactin and rhamnolipid extracts, produced by ST34 and ST5, respectively, were analysed against various actively growing reference (ATCC), environmental and clinical Gram-positive and Gram-negative bacterial strains (Table 1) as well as fungal strains (Table 2). This was achieved by utilising an agar disc susceptibility modified method as outlined in Das et al. (2008). The zones of inhibition produced by each biosurfactant extract against each microbial strain used as a test organism, were recorded.

### Antimicrobial activity of ST34 extract

The extracts of strain ST34 were tested against Gram-negative reference (ATCC) strains (n = 10), as well as environmental (n = 8) and clinical (n = 2) strains. Overall, antibacterial activity was observed against all the Gram-negative bacteria (100%) analysed as test organisms (Table 1), with varying diameters for the zones of inhibition recorded. For the ten Gram-negative reference strains, the ST34 extracts displayed the lowest zone of inhibition of 10 mm against *E. coli* ATCC 13706 and the highest zone of inhibition against *Salmonella typhimurium* ATCC 14028 at 25.3 ± 1.2 mm (Table 1). An average zone of inhibition of 15.2 ± 0.6 mm was observed for the reference strains. The ST34 extract was also tested against environmental strains (Table 1), with the smallest zone of inhibition (9.8 ± 0.8 mm) observed against the *Serratia* sp. L8 strain and the largest zone of inhibition (17.7 ± 1.9 mm) observed against the *E. coli* K4CCA strain. An average zone of inhibition of 13 ± 0.6 mm was observed for all environmental strains. Furthermore, the ST34 extracts displayed zones of inhibition of 13 ± 0.8 and 13.3 ± 0.2 mm against the two clinical *K. pneumoniae* strains (P2 and P3), respectively. It should be noted that while the antimicrobial assays were conducted against the test bacterial strains utilising the 15% methanol surfactin (crude extract) extract, the antimicrobial assays conducted utilising the 70% acetonitrile surfactin extract against *E. coli* ATCC 13706 produced similar results. The antimicrobial activity (12.5 ± 0.9 mm) of the 15% methanol surfactin and the 70% acetonitrile surfactin extract against *E. coli* ATCC 13706 were thus comparable. The antibacterial activity of the commercial pure surfactin against *E. coli* ATCC 13706 (12.7 ± 1.2 mm) was also comparable to the antibacterial activity of both surfactin extracts (15% methanol and 70% acetonitrile extracts) obtained from *B. amyloliquefaciens* ST34.

The ST34 extract was then tested against Gram-positive reference strains (n = 3), as well as environmental (n = 5) and clinical (n = 3) strains. Overall, antibacterial activity was observed against 90.1% of the Gram-positive bacteria analysed as test organisms (Table 1), with varying diameters for the zones of inhibition recorded. All the reference strains displayed sensitivity against the extract produced by ST34, where the smallest zone of inhibition (10.3 ± 0.5 mm) was observed for *B. cereus* ATCC 10876 and the largest zone of inhibition (14.7 ± 0.5 mm) was observed for *Staphylococcus aureus* ATCC 25923. An average zone of inhibition of 12.7 ± 0.6 mm was observed for the reference strains. For the five environmental strains utilised, the surfactin extract displayed no zone of inhibition against *B. cereus* ST18, while the largest zone of inhibition (14 mm) was obtained against *Micrococcus* sp. AQ4S2. An average zone of inhibition against environmental Gram-positive bacterial strains was observed at 10 ± 0.2 mm. The ST34 extract was also tested against the clinical strains, which displayed the smallest zone of inhibition of 15.3 ± 0.5 mm against *S. aureus* (MRSA) Xen 30, while the largest zone of inhibition was observed at 18.7 ± 0.9 mm against *E. faecalis S2*. An average zone of inhibition of 17.4 ± 0.9 mm was observed for the clinical strains. The antibacterial activity of the commercial pure surfactin against *S. aureus* ATCC 25923 (17.8 ± 0.8 mm) was also comparable to the crude surfactin extract obtained from *B. amyloliquefaciens* ST34.

Five clinical and five environmental fungal strains were utilised as test organisms for the antimicrobial assessment of solvent extracted from ST34 (Table 2). The ST34 extract exhibited pronounced antifungal activity against 80% (4/5) of the clinical strains tested. No antifungal activity was observed against *Cryptococcus neoformans* 1063, while the largest zone of inhibition of 15.3 ± 0.5 mm was observed for C. *neoformans* CAB1055. An average zone of inhibition of 10.7 ± 0.4 mm was observed for the clinical strains. The ST34 extract also displayed antifungal activity against 60% (3/5) of the environmental fungal isolates utilised in the current study. No zone of inhibition was observed for *C. neoformans* CAB1034 and *Candida albicans* 1085 and the largest zone of inhibition of 15.3 ± 1.2 mm was observed for the *Cryptococcus neoformans* CAB844 environmental strain. An average zone of inhibition of 7.9 ± 0.8 mm was observed for the environmental fungal strains. In addition, the antifungal activity of the commercial pure surfactin against *C. neoformans* CAB1055 (13.7 ± 1.2 mm) was comparable to the surfactin extract obtained from *B. amyloliquefaciens* ST34.

### Antimicrobial activity of ST5 extract

The extract of strain ST5 was tested against the Gram-negative reference (ATCC) (n = 10), environmental (n = 8) and clinical (n = 2) strains. Overall, antibacterial activity was observed against all the Gram-negative bacterial (100%) strains analysed as test organisms (Table 1), with varying diameters for the zones of inhibition recorded. For the reference strains, the ST5 extract displayed the smallest zone of inhibition (13 mm) against Enterotoxigenic *E. coli* H10407, while the largest zone of inhibition (29.3 ± 0.9 mm) was observed against *E. coli* ATCC 13706. An average zone of inhibition of 18.5 ± 0.7 mm was obtained against the reference strains. For the environmental strains, the ST5 extract produced the smallest zone of inhibition of 9.8 ± 0.8 mm against the *Serratia* sp. L8 strain, while the largest zone of inhibition of 17.7 ± 1.9 mm was recorded against *E. coli* K4CCA. The average zone of inhibition against the environmental strains was 13.6 ± 0.9 mm. Furthermore, the ST5 extracts displayed zones of inhibition of 8.3 ± 0.5 and 11.7 ± 0.9 mm against the two clinical *K. pneumoniae* strains (P2 and P3), respectively. For the antimicrobial assays conducted against *E. coli* ATCC 13706 utilising the 15% methanol rhamnolipid (crude extract) extract and the 70% acetonitrile rhamnolipid extract, a decreased antimicrobial activity was observed for the 70% acetonitrile rhamnolipid extract (15.3 ± 1.5 mm) in comparison to the crude extract. In addition, the antibacterial activity of the commercial pure rhamnolipid against *E. coli* ATCC 13706 (13.3 ± 1.2 mm) was lower in comparison to both rhamnolipid extracts (15% methanol and 70% acetonitrile extracts) obtained from *P. aeruginosa* ST5.

The ST5 extract was also tested against Gram-positive reference (n = 3), environmental (n = 5) and clinical (n = 3) strains. Overall, antibacterial activity was observed against all the Gram-positive bacterial (100%) strains analysed as test organisms (Table 1), with varying diameters for the zones of inhibition recorded. For the reference strains, the smallest zone of inhibition of 13.0 ± 0.8 mm was recorded for *B. cereus* ATCC 10876, while the largest zone of inhibition of 17 ± 1.4 mm was recorded for *B. cereus* LMG 13569. An average zone of inhibition of 14.6 ± 0.9 mm was obtained. For the Gram-positive environmental strains, the smallest zone of inhibition of 11 mm was recorded for *S. aureus* C3, while the largest zone of inhibition (22.3 ± 0.9 mm) was observed against *B. cereus* ST18. An average zone of inhibition of 15.4 ± 0.9 mm was obtained against the environmental Gram-positive strains. The ST5 extract also displayed activity against all clinical strains, with the smallest zone of inhibition of 10.7 ± 0.5 mm recorded for *E. faecalis* S1 and the largest zone of inhibition of 21.7 ± 2.4 mm recorded for *E. faecalis* S2. The average zone of inhibition produced by the ST5 extract against the clinical strains was 15.2 ± 1.1 mm. The antibacterial activity of the commercial pure rhamnolipid against *S. aureus* ATCC 25923 (13.3 ± 1.2 mm) was then comparable to the antibacterial activity of the rhamnolipid extract obtained from *P. aeruginosa* ST5.

Five clinical and five environmental fungal strains were utilised as test organisms for the antimicrobial assessment of solvent extracted compounds from ST5 (Table 2). The ST5 extract displayed antifungal activity against 100% (5/5) of the clinical strains tested. The smallest zone of inhibition of 11.3 ± 0.9 mm was observed for *C. neoformans* CAB 1055 and the largest zone of inhibition (14.7 ± 0.5 mm) was obtained against *C. albicans* 8911 strain. An average zone of inhibition by the ST5 extract against the clinical strains was recorded as 13 ± 1.2 mm. The ST5 extract then displayed 80% (4/5) antifungal activity against the environmental fungal strains. No zone of inhibition was observed against *C. neoformans* CAB842, and the largest zone of inhibition (18 ± 0.8 mm) was observed against *C. neoformans* 1034. An average zone of inhibition of 12 ± 1 mm was observed for the ST5 extract against the environmental fungal strains. The antifungal activity of the commercial pure rhamnolipid against *C. neoformans* CAB1055 (12.7 ± 1.2 mm) was comparable to the antifungal activity of the rhamnolipid extracts obtained from *P. aeruginosa* ST5.

## Discussion

Bacteria, fungi and yeast producing biosurfactant compounds, which display broad spectrum antimicrobial properties, are usually isolated from diverse terrestrial environments such as the rhizosphere, contaminated soils and hydrocarbon polluted water sources (Bento et al. 2005; Pornsunthorntawee et al. 2008). Initial analysis then indicated that the two bacterial strains ST34 (*B. amyloliquefaciens*) and ST5 (*P. aeruginosa*) isolated from wastewater, produced biosurfactants (Ndlovu et al. 2016). The current study thus focused on the partial purification and characterisation of the antimicrobial lipopeptide and glycolipid biosurfactant compounds produced by ST34 and ST5, respectively. The extracts obtained from the ST34 and ST5 cultures were characterised using a method that was developed in the current study for use with the UPLC–MS analysis, which facilitated the successful detection and separation of different analogues of the surfactin (ST34) and rhamnolipids (ST5) produced by the respective strains.

The solvent extracts obtained from the *B. amyloliquefaciens* (ST34) strain were confirmed to contain surfactin lipopeptides, in which the structural surfactin analogues with a mass difference of 14 or 28 amu. These differences are consistent with a CH_2_ moiety correlating either to a Val to IIe/Leu modification or longer/branched fatty acyl chain (CH_2_–CH_2_ moiety). The UPLC–MS separation successfully differentiated between the surfactin analogues in the same mixture, which were identified as C_13_, C_14_, C_15_ and C_16_ surfactin analogues (Srf1-5 groups) (Table 3). The different groups were observed to have two or more retention times even though they displayed the same *m/z* and *M*_r_ values. The lle containing peptides possibly elute at a slightly different R_t_ to those substituted with Leu due to the slight differences in their hydrophobicity (Yang et al. 2015), for example Srf4 eluted at 12.1 and 12.2 min (Table 3). Within each of the five surfactin groups obtained for the ST34 strain, two or more surfactin analogues were detected. The Srf4 group was the most dominant with a relative abundance of approximately 60% in the ST34 NA culture extracts, while the Srf3 and Srf4 groups were observed at approximately 43 and 33%, respectively, in the glycerol-MSM culture extracts. Results obtained in the current study are comparable to a study conducted by Pecci et al. (2010), were they successfully identified different surfactin (C_13_ (Srf1-2), C_14_ (Srf2-3) and C_15_ (Srf4) surfactins), fengycin A and B analogues compounds produced by *Bacillus licheniformis* V9T14. The authors utilised the LC–ESI–MS/MS for the separation and partial characterisation of the surfactin analogues and fengycin isoforms, as well as the relative percentage content of each compound.

The solvent extracts obtained from the ST5 strain were confirmed to be a mixture of rhamnolipid congeners of monorhamnolipids (Rha–C_12_–C_10_/Rha–C_10_–C_12_; Rha–C_10_–C_10_; Rha–C_10_–C_8_/Rha–C_10_–C_8_) and dirhamnolipids (Rha–Rha–C_12_–C_10_/RhaRha–C_10_–C_12_; Rha–Rha–C_10_–C_10_; Rha–Rha–C_10_–C_8_/Rha–Rha–C_10_–C_8_). These results are in agreement with a study conducted by Pantazaki et al. (2011), where similar rhamnolipid congeners were detected. Additionally, the detected HAAs in the current study could either be intermediates in rhamnolipid biosynthesis or rhamnolipid fragments obtained by cleavage in the ESI–MS of the rhamnosyl group (hydrophilic moiety) (Lépine et al. 2002). A study conducted by Pereira et al. (2012) on rhamnolipids produced by *P. aeruginosa* strains also illustrated that MS coupled with electrospray ionisation provided an accurate and rapid characterisation of the monorhamnolipids [Rha–C_10_–C_10_, Rha–C_10_–C_12_, Rha–C_10_–C_12:1_] and dirhamnolipids [Rha–Rha–C_10_–C_10_, Rha–Rha–C_10_–C_12_]. Itoh et al. (1971) then produced a mixture of monorhamnolipid (Rha–C_10_–C_10_) and dirhamnolipid (Rha–Rha–C_10_–C_10_) congeners using the *P.* *aeruginosa* KY 4025 strain, which they purified and separated using the HPLC based method to obtain individual rhamnolipids. Moreover, they showed that individual rhamnolipids (Rha–C_10_–C_10_ and Rha–Rha–C_10_–C_10_) displayed pronounced antibacterial activity against both Gram-negative and Gram-positive strains, including a multi-drug resistant *E. coli* strain.

The antimicrobial activity of the extracts containing surfactin and rhamnolipid congeners produced by ST34 and ST5 against various reference, environmental and clinical bacterial and fungal strains was then determined. Results indicated that both extracts displayed 100% antibacterial activity against the Gram-negative bacteria analysed (Table 1). However, based on the average zones of inhibition, the surfactin extract (ST34) exhibited an increased antibacterial activity against the clinical strains (average zone of inhibition of 13.2 ± 0.5 mm), while the rhamnolipid extract (ST5) produced noticeable activity (average zone of inhibition of 18.5 ± 0.7 mm) against the reference target strains. No significant difference between the surfactin and rhamnolipid extract’s antibacterial activity against the Gram-negative environmental (p = 0.58) and reference (p = 0.17) bacterial strains, respectively, was however observed. In addition, the surfactin extract displayed a higher antibacterial activity against the Gram-positive clinical strains (average zone of inhibition 17.4 ± 0.9 mm), while the rhamnolipid extracts produced an increased antibacterial activity against the Gram-positive environmental (average zone of inhibition 15.4 ± 0.5 mm) and reference strains (average zone of inhibition 14.6 ± 0.9 mm). However, the two tailed t-test showed that there was no significant difference between the zones of inhibition obtained against the clinical (p = 0.56) and environmental (p = 0.12) Gram-positive strains, respectively for the surfactin and rhamnolipid extracts. Moreover, the surfactin and rhamnolipid extracts displayed no significant difference (p = 0.34) between the zones of inhibition obtained against the reference Gram-positive strains. Of particular interest was the sizeable zone of inhibition (22.3 ± 0.9 mm) recorded for the rhamnolipid extract against the *B. cereus* ST18, which was seemingly resistant to the surfactin extract as no zone of inhibition was recorded.

Research has indicated that approximately 5% of the genome of most *Bacillus* species encodes for the synthesis of antimicrobial compounds (Stein 2005). Of these structurally diverse antimicrobial compounds, approximately two dozen have been characterised, with the cyclic lipopeptides of three families fengycin, iturin and surfactin displaying antifungal and antibacterial properties (Mandal et al. 2013). Surfactin exhibits an antimicrobial mechanism by accumulating on the surface of the microbial cell (bacteria and fungi) until a threshold concentration is reached. Thereafter they permeate the membrane leading to its disintegration by a detergent-like mechanism (Yao et al. 2012). This disintegration is hypothesised to occur by the formation of pores in the cell membrane of microbial cells thus inducing an increased influx of Ca^2+^ and H^+^ into the cells (Thrane et al. 1999). Comparatively, rhamnolipids have structures and properties similar to that of detergents and have been reported to intercalate into the membrane phospholipid bilayer thereby facilitating the permeability of the membrane and flow of metabolites (Sotirova et al. 2008). The structure and function of the phospholipid bilayer is thus altered, effectively interrupting protein conformation, transport and energy generation, which eventually leads to cell death.

It should be noted that of the 31 bacterial strains analysed in the current study, three strains were resistant to various classes of antibiotics [Enteropathogenic *E. coli* B170 resistant to gentamicin, *S. aureus* ATCC 25923 resistant to oxacillin, *S. aureus* Xen 30 resistant to methicillin, gentamicin, oxacillin and tetracycline (Table 1)]. The results obtained in the current study indicated that these strains were sensitive to both the surfactin and rhamnolipid extracts produced by ST34 and ST5, respectively. Moreover, 90% of the fungal strains analysed in the current study were susceptible to the rhamnolipid extract, while only 70% of the fungal strains were susceptible to the surfactin extract. Although, after performing a two-tailed t-test analysis, no significant difference (p = 0.183) between the zone of inhibition of surfactin and rhamnolipid extracts against the fungal strains analysed was obtained. Yoshida et al. (2001), then showed that the cell free supernatant (containing surfactin) of *B. amyloliquefaciens* RC-2, isolated from healthy Mulberry leaves, strongly inhibited the growth of 44 and 40% of bacteria and fungi isolates, respectively. In a study conducted by Sun et al. (2006), a *B.* *amyloliquefaciens* ES-2 isolate was also shown to produce antimicrobial lipopeptide compounds (fengycins and surfactins), which demonstrated antimicrobial activity against a total of 37 microorganisms (including *E. coli*, *S. aureus* and *B. cereus*). In a study conducted by Abalos et al. (2001), a rhamnolipid mixture that consisted of Rha–C_10_–C_10_, Rha–C_10_–C_12_, Rha–Rha–C_10_–C_10_, Rha–Rha–C_10_–C_12_, then displayed broad spectrum antimicrobial activity against a wide range of organisms, including *C. albicans, S. marcescens, B. cereus* and *S. aureus* strains. However, in a previous study conducted by Liu et al. (2012), it was demonstrated that the surfactin C_15_ analogue together with ketoconazole (a synthetic antifungal compound) exhibited effective synergistic antifungal activity against *C. albicans* SC5314 at concentrations of >6.25 and 0.004 µg mL^−1^, respectively. These concentrations were lower than the individual antifungal activity observed at >100 µg mL^−1^ (surfactin C_15_) and 0.016 µg mL^−1^ (ketoconazole) (Liu et al. 2012). Future studies on the possible synergistic effects of other compounds on the antimicrobial activity of the surfactin and rhamnolipid extracts produced by ST34 and ST5 strains, respectively, will thus be highly beneficial to elucidate the role of each compound in the observed antimicrobial activity.

In the current study, the optimised UPLC–MS method was successfully employed to characterise the extracted surfactin and rhamnolipid mixtures produced by the *B. amyloliquefaciens* ST34 and *P. aeruginosa* ST5 isolates in liquid and on agar media. The *B. amyloliquefaciens* ST34 strain produced a mixture of surfactin analogues (Srf1-5), which have a synergistic effect on inhibiting bacterial and fungal growth. The most abundant surfactin groups were Srf4 > Srf3 > Srf2 with minor contributions by Srf1 and Srf5. The Rha–C_10_–C_10_ and Rha–C_10_–C_8_ or Rha–C_10_–C_8_ were the most abundant monorhamnolipids in the extracts, while the Rha–Rha–C_10_–C_10_ and Rha–Rha–C_10_–C_8_ or Rha–Rha–C_10_–C_8_ were the most abundant dirhamnolipids produced by the *P. aeruginosa* ST5 strain. In this context, the results indicate that our rapid extraction and UPLC–MS method can be a simple and powerful technique to provide fast, sensitive and accurate identification of a variety biosurfactant compounds synthesised by microbial strains. In addition, pronounced antimicrobial activity against diverse microorganisms, including antibiotic resistant *S. aureus* and *E. coli,* as well as the fungal pathogens *C. albicans* and *C. neoformans* was retained by both the surfactin and rhamnolipid extracts. The two biosurfactant producing strains isolated from wastewater thus show potential for large-scale production of various analogues/congeners of the surfactin and rhamnolipid biosurfactant compounds for utilisation in the medical and food industries as antimicrobial agents.

## Additional file

**Additional file 1: Figure S1.** Comparison of the different culture extracts showing the relative contribution of each of the surfactin groups in the biosurfactant extracts. The contribution was calculated from UPLC profiles, with the assumption that all the surfactin species has similar ion responses. SARCC 697 (ST34) LC = glycerol-MSM culture extract, SARCC 697 (ST34) LC-AE = 70% acetonitrile extract of SARCC 697 (ST34) LC, SARCC 697 (ST34) NA-TSC = NA test tube slant culture extract, SARCC 697 (ST34) NA-PDC = NA petri dish culture extract.

## Abbreviations

[M+H]^+^: singly charged protonated ion specie
[M+Na]^+^: singly charged sodium adduct
amu: atomic mass unit
ATCC: American Type Culture Collection
Da: daltons
dRL: dirhamnolipid
ESI–MS: electrospray ionisation mass spectrometry
HAAs: 3-(3-hydroxyalkanoyloxy) alkanoic acids
HCl: hydrochloric acid
LB: Luria Bertani
MHA: Mueller–Hinton agar
*M*_*r*_: relative molecular mass
mRL: monorhamnolipid
MSM: mineral salt medium
NA: nutrient agar
Rha–C_8_–C_10_: α-l-rhamnopyranosyl-β-hydroxyoctanonyl-β-hydroxydecanoate
Rha–C_10_–C_8_: α-l-rhamnopyranosyl-β-hydroxydecanoyl-β-hydroxyoctanoate
Rha–C_10_–C_10_: α-l-rhamnopyranosyl-β-hydroxydecanoyl-β-hydroxydecanoate
Rha–Rha–C_8_–C_10_: α-l-rhamnopyranosyl-α-l-rhamnopyranosyl-β-hydroxyoctanonyl-β-hydroxydecanoate
Rha–Rha–C_10_–C_8_: α-l-rhamnopyranosyl-α-l-rhamnopyranosyl-β-hydroxydecanoyl-β-hydroxyoctanoate
Rha–Rha–C10–C10: α-l-rhamnopyranosyl-α-l-rhamnopyranosyl-β-hydroxydecanoyl-β-hydroxydecanoate
RL: rhamnolipid
SARCC: South African Rhizobium Culture Collection
Srf: surfactin
UPLC–MS: ultraperformance liquid chromatography coupled with mass spectrometry
