# Supplementary material for: Characterisation and antimicrobial activity of biosurfactant extracts produced by Bacillus amyloliquefaciens and Pseudomonas aeruginosa isolated from a wastewater treatment plant
Source: AMB Express. 2017 May 31;7:108. doi: 10.1186/s13568-017-0363-8 (PMC5451374; doi:10.1186/s13568-017-0363-8)
Supplement: Supplementary file 1 — Additional file 1: Figure S1. Comparison of the different culture extracts showing the relative contribution of each of the surfactin groups in the biosurfactant extracts. The contribution was calculated from UPLC profiles, with the assumption that all the surfactin species has similar ion responses. SARCC 697 (ST34) LC = glycerol-MSM culture extract, SARCC 697 (ST34) LC-AE = 70% acetonitrile extract of SARCC 697 (ST34) LC, SARCC 697 (ST34) NA-TSC = NA test tube slant culture extract, SARCC 697 (ST34) NA-PDC = NA petri dish culture extract. [file 13568_2017_363_MOESM1_ESM.pdf]

AMB Express
SUPPLEMENTARY MATERIAL

Characterisation and antimicrobial activity of biosurfactant extracts produced by *Bacillus amyloliquefaciens* and *Pseudomonas aeruginosa* isolated from a wastewater treatment plant

Thando Ndlovu[1], Marina Rautenbach[2], Johann Arnold Vosloo[2], Sehaam Khan[3] and Wesaal Khan[1*]

[1]Department of Microbiology, Faculty of Science, Stellenbosch University, Private Bag X1, Stellenbosch, 7602, South Africa.

[2] BIOPEP Peptide Group, Department of Biochemistry, Faculty of Science, Stellenbosch University, Private Bag X1, Stellenbosch,7602, South Africa.

[3]Faculty of Health and Applied Sciences, Namibia University of Science and Technology, 13 Storch Street, Private Bag 13388, Windhoek, Namibia.

*Corresponding Author: Wesaal Khan, Email: wesaal@sun.ac.za, Tel: +27 21 808 5804, Fax: +27 21 808 5846

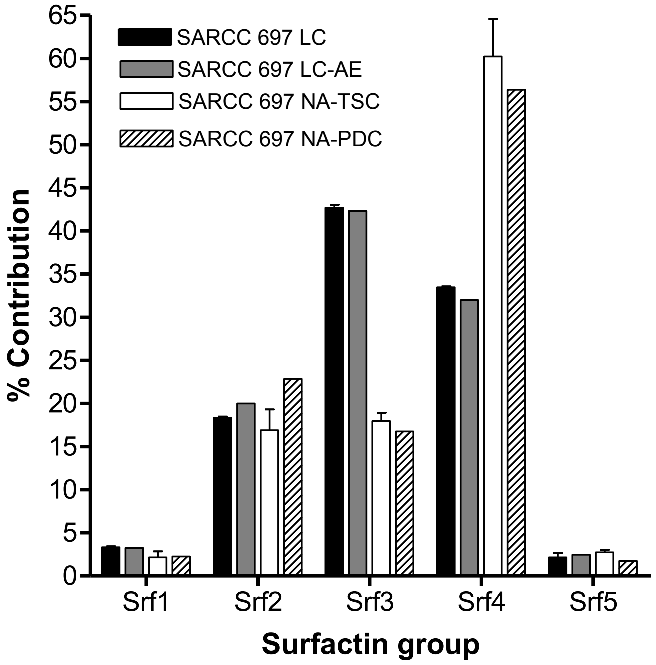

Fig. S1 Comparison of the different culture extracts showing the relative contribution of each of the surfactin groups in the biosurfactant extracts. The contribution was calculated from UPLC profiles, with the assumption that all the surfactin species has similar ion responses. SARCC 697 LC = glycerol-MSM culture extract, SARCC 697 LC-AE = 70% acetonitrile extract of SARCC 697 LC, SARCC 697 NA-TSC = NA test tube slant culture extract, SARCC 697 NA-PDC = NA petri dish culture extract
